# Some Experimental and Clinical Observations upon the Therapeutical Value of Salicylbromanilid

**Published:** 1891-07

**Authors:** C. S. Bradfute

**Affiliations:** Demonstrator of Therapeutics, Jefferson Medical College, Philadelphia


### SOME EXPERIMENTAL AND CLINICAL OBSERVATIONS UPON THE THERAPEUTICAL VALUE OF SALICYLBROMANILID.

By C. S. BRADFUTE, M. D.,

Demonstrator of Therapeutics, Jefferson Medical College, Philadelphia.

Among the new remedies lately introduced from Germany is one from Radlauer's laboratory, a synthetical compound, to which he has given the name "antinervin," or, with a view of indicating its chemical composition, salicylbromanilid. The former is its proprietary title. It is a combination of bromacetanilid and salicylanilid, and is claimed to possess the virtues of antifebrin, bromine and salicylic acid, without their unpleasant effects, and is, consequently, an antipyretic, an antineuralgic, and an antinervine. It is a white, crystaline powder, having a rather pleasant, slightly acid taste, feebly soluble in cold water, but dissolves freely in hot water, alcohol or ether. The dose is from three to ten grains, and is best given in the form of compressed tablets or in simple powders. The writer takes the liberty of suggesting that its chemical name be abbreviated, as it seems unnecessarily long; it could be easily called "salbromalid," which would accomplish the object of brevity, and, at the same time, sufficiently indicate the chemical nature of the compound.

A glance at the physiological action of the three agents comprising salicylbromanilid, shows that they are essentially circulatory depressants. Salicylic acid acts directly on the heart muscle, lessening its electro-contractility, and, when administered in toxic doses, causing the organ to stop in diastole. After a preliminary period of stimulation, it depresses the vaso-motor centers. Antifebrin acts very similarly, though its effect upon the heart and vessels is more powerful, producing a rapid fall in the blood-pressure and a weak, irregular heart. Bromine, in addition to its impression upon the heart and vaso-motor nervous system, lowers the vital activity of the centers in the *medulla oblongata*, and interferes with the function of conscious cerebration in a way not yet clearly understood.

It can thus be seen that a compound made up of these three substances, when given in full physiological doses, would probably exhibit an action upon the system manifested by a profound interference with the motor mechanism of the circulatory apparatus, and that whatever therapeutical value could be attached to it from a pharmacological standpoint, would depend upon this action.

In a series of experiments conducted in the therapeutical laboratory in the Jefferson Medical College, the writer's observations were confirmatory of the above remarks. He found antinervin a profound depressant of the circulation, and a prompt antipyretic. Three grains injected into the lymph sac of a medium-sized frog produced death in one hour without convulsions, the animal becoming languid and indifferent to mild stimulation after the lapse of ten minutes, and passing rapidly into stupor, finally died in a condition of coma with the muscular system completely relaxed. The reflexes gradually diminished during the course of the poisoning and were totally absent eight minutes before the cessation of the circulation.

A similar quantity was injected into a frog so prepared that the movements of the heart could be observed *in situ* and the capillary circulation watched under the microscope. The cardiac cycle was observed to gradually and uniformly become longer, the contractions lessened in vigor, the ventricles contracted more slowly than the auricles, reacting lazily to an electric current, and finally the heart stopped in diastole, spreading out like mush when removed from the body and placed upon a glass plate. The capillaries dilated, slowly and irregularly at first, but fifteen minutes before death relaxed entirely, and the blood current diminished in rapidity in proportion to the capillary paresis and the cardiac depression, the corpuscles tumbling along against each other and showing a tendency to adhere to the vessel wall. Death occurred in forty-six minutes.

The behavior of the heart in the above experiment indicated the poisonous effect of the drug directly upon the organ, but, in order to prove this, the heart of a healthy bactrachian was taken out of the body and placed in a Kronecker-Bowditch apparatus. Here, removed from the influence of the central nervous system, a solution of antinervin was permitted to flow, by means of a perfusion canula introduced into the ventricle, slowly through the heart, and the results observed were the same as those noted when the heart was *in situ*. A control experiment eliminated any undue influences upon the heart from the damage it sustained in placing it in the apparatus.

Upon the rabbit the drug acts very much the same as upon the cold-blooded animal, and its influence over the respiratory movements, which is more distinct in warm-blooded animals, shows the part played by the salicylic acid in the general result. Respiration became rapid, weak, shallow, and stopped before the heart, the latter becoming slower and more feeble, and finally, a few minutes before the circulation ceased, would make no impression upon the drum of a cardiograph.

Guided by these experiments, the writer concluded that salbromalid was best applicable to those affections characterized by functional disturbances of the circulatory system brought about by reflex impressions or too active stimulation, and acute inflammatory conditions occurring in robust subjects. In the cases that fell in his hands he found this conclusion correct, and noted favorable results, and, in some instances obtained, curative effects when other remedies had failed or acted unsatisfactorily.

The following are a few of the cases in which he employed the remedy, and, while they are not conclusive in establishing the therapeutical position of the drug, they may be accepted as indications for its administration :

Case I. Angina pectoris. Male, aged 36 ; laborer. Has attacks of angina pectoris about twice a month. During paroxysm face is pale, extremities cold, arterial tension high, and pain so excruciating as to cause at times symptoms resembling acute mania. Ten grains of salbromalid caused relief of symptoms in about twenty minutes, and three grains every two hours afterwards prevented a recurrence of the paroxysm. The results were, of course, not permanent, as the patient still has attacks as frequently as ever, but the drug never fails to check a paroxysm. The writer enjoins a caution here in administering this drug in angina pectoris. It should not be given in asthenic cases, as there must always be at hand ammonia and strychnine to combat a failure in the circulation. A thirtieth of a grain of the latter, hypodermatically, if the heart shows signs of ceasing work, is the proper dose.

Case III. Typhoid fever, in second week. Male, aged 23 ; clerk. Temperature, 104.4° F. ; pulse, 100 ; respiration, 24. Five grains of salbromalid reduced the temperature to 102.3° F. within one hour and a half. No bad results followed.

Only one dose was administered to this case, as cold-sponging was sufficient to retain the temperature within safe limits, and it was not deemed advisable to tamper with a weak typhoid circulation.

Case III. Brachial neuralgia of two weeks' duration. Female, aged thirty-two ; type-writer. Pain paroxysmal. Three grains of salbromalid, administered every three hours, caused the pain to disappear within twelve hours. This dosage was continued four days, and afterwards a course of arsenic and diet effected a permanent cure.

This patient was robust, but of a neurotic temperament, and the neuralgic pain was evidently spasmodic in character. The following case presented the converse condition, and it will be noticed that the drug was ineffective.

Case IV. Brachial neuralgia of three years' duration, probably rheumatic. Man, aged forty-one; engineer. In fair physical health, with a rather stolid, morose disposition. Suffers more or less continuous dull pains in left axillary and brachial regions, with occasional exacerbations. Ten grain doses of salbromalid depressed the circulation but exercised no appreciable control over the pain.

Case V. Acute inflammatory rheumatism. Female, aged thirty-seven; cook. Temp. 104° F.; pulse 108; resp. 26. Five grains of salbromalid reduced the temperature to 103° F., and diminished the general sense of discomfort and uneasiness. It was repeated in four hours, with the result of further reducing the temperature, but, also, of markedly depressing the circulation, and it was not again administered, as the patient developed pericarditis in a severe form on the fifth day. In this case the remedy would, undoubtedly, have acted better if it had been given in smaller doses.

Radlauer claims antinervin to be anti-diabetic, but in one case of diabetes, in which the writer had an opportunity of employing it, no diminution was observed in the amount of water and sugar excreted, but, of course, one trial cannot be accepted as conclusive evidence of its inutility in this affection.

It is seen from what has been stated, that salbromalid is most effective as a pain reliever and antinervine in those functional disturbances of the circulatory system which occur at the onset of acute diseases, and in some other conditions, manifested by an overacting heart and contraction of the arterioles, which lessens the total area of blood space, and that it is most effective in robust subjects. Its power to reduce the temperature is undoubted, but owing to its action upon the heart it should be given carefully in states of hyperpyrexia, especially the low fevers.—*New England Medical Monthly.*

